# Dataset on antioxidant metabolites and enzymes activities of freshly harvested sweet cherries (*Prunus avium* L.) of Campania accessions

**DOI:** 10.1016/j.dib.2017.10.020

**Published:** 2017-10-14

**Authors:** Antonio Mirto, Federica Iannuzzi, Petronia Carillo, Loredana F. Ciarmiello, Pasqualina Woodrow, Amodio Fuggi

**Affiliations:** Dipartimento di Scienze e Tecnologie Ambientali, Biologiche e Farmaceutiche, Università degli Studi della Campania “Luigi Vanvitelli”, Via Vivaldi 43, Caserta, Italy

## Abstract

In this article, we reported the original data obtained by the study of metabolites and enzymes involved in sweet cherry antioxidant system. We measured hydrogen peroxide (H_2_O_2_) and malondialdehyde (MDA), which are indicator of oxidative stress. Moreover, we measured the concentration of reduced and oxidized ascorbate and glutathione that are involved in ROS detoxification together with phenolics, anthocyanins and tocopherols. Among antioxidant enzymes, we analyzed the activities of ascorbate peroxidase (APX; EC 1.11.1.11), and the soluble and bound forms of polyphenol oxidase (PPO; EC 1.10.3.1) and guaiacol peroxidase (POD; EC 1.11.1.7). The data reported in this paper are related to the research article “Metabolic characterization and antioxidant activity in sweet cherry (Prunus avium L.) Campania accessions”, authored by Mirto et al. (2018) [1].

**Specifications Table**

**Table:** 

Subject area	*Agricultural and biological science*
More specific subject area	*Postharvest physiology*
Type of data	*Tables*
How data was acquired	*Spectrofluorometric detection (Synergy HT, Biotek), DAD/UV and FLD HPLC detection (HP-1100, Agilent)*
Data format	*Raw data statistically analyzed*
Experimental factors	*The data concern freshly harvested cherries without any pretreatment*
Experimental features	*The experimental design included metabolic profiling and enzyme activity analyses*
Data source location	*Eboli (SA), Campania, Italy. 40 °33′N, 14 °58′E*
Data accessibility	*Mean and standard deviation of data are available in this article, raw data are available at* https://www.dropbox.com/s/fhohq32zzhx0f2e/DIB-D-17–00711.xlsx?dl=0
Related research article	*This article is submitted as a companion paper* to Mirto et al. (2018) [1]

**Value of the data**

• The data show the antioxidant metabolites and enzyme activities measured in sweet cherries fruits.
• The data highlight the differences among the different sweet cherries Campania accessions.
• The data are useful to identify the accessions more suitable for long-term storage.

## 1 Data

Selecting fruit accessions rich in antioxidant can help delaying fruit senescence and better preserving its characteristics during postharvest. To this aim, we investigated antioxidant metabolites level and antioxidant enzyme activities in forty-three accessions of sweet cherry fruits from Campania region [1].

In Table 1 we reported the concentration of hydrogen peroxide (H_2_O_2_), as well as malondialdehyde (MDA) of sweet cherry fruit accessions. MDA is considered a useful index of general lipid peroxidation and a biomarker for oxidative stress [2]. In Table 1 reduced and oxidized forms of ascorbate (AsA and DHA) and glutathione (GSH and GSSG) which are indicators of oxidative stress were also presented. A high reduced per oxidized ratio of the two metabolites is essential for ROS scavenging in plant cells [3], [4], [5]. In Table 2 we showed the concentration of phenolics, anthocynanins and tocopherols, involved in ROS detoxification together with ascorbate and glutathione [3], [4].

**Table 1:** Hydrogen peroxide (H_2_O_2_), malondialdehyde (MDA), reduced and oxidized ascorbate (AsA and DHA)) and glutathione (GSH and GSSG) contents in sweet cherry fruits. All data are expressed as mg 100 g^-1^ FW ± SD (n=3).

**Accession**	**H**_**2**_**O**_**2**_			**MDA**			**AsA**			**DHA**			**GSH**			**GSSG**		
1 - Antuono	2.54	±	0.07	0.25	±	0.02	9.90	±	1.10	1.90	±	0.40	38.5	±	3.9	2.70	±	0.60
2 - Bertiello	2.76	±	0.38	0.35	±	0.06	7.30	±	0.00	2.50	±	0.90	12.6	±	2.3	1.50	±	0.10
3 - Bologna	1.47	±	0.35	0.29	±	0.01	10.70	±	0.20	4.00	±	0.60	46.8	±	3.6	1.40	±	0.10
4 - Camponica	2.01	±	0.13	0.12	±	0.02	8.10	±	0.70	2.10	±	0.70	21.3	±	3.8	1.60	±	0.00
5 - Cannamela	2.35	±	0.13	0.20	±	0.02	12.50	±	1.10	2.00	±	0.80	47.8	±	6.5	2.00	±	0.10
6 - Casanova	2.30	±	0.38	0.30	±	0.05	4.10	±	1.00	11.00	±	0.90	32.7	±	7.8	1.70	±	0.10
7 - Cervina	2.81	±	0.30	0.18	±	0.03	15.50	±	1.40	2.70	±	1.00	70.2	±	2.0	1.80	±	0.30
8 - Cervone	2.38	±	0.26	0.22	±	0.02	19.20	±	2.60	3.60	±	0.40	47.7	±	11.4	1.50	±	0.10
9 - Ciauzara	2.70	±	0.09	0.02	±	0.01	5.50	±	1.10	7.30	±	0.80	16.3	±	3.1	2.00	±	0.10
10 - Corniola	2.28	±	0.15	0.14	±	0.01	2.00	±	0.40	1.00	±	0.20	75.8	±	7.8	1.70	±	0.30
11 - Corvina	3.27	±	0.23	0.31	±	0.06	10.40	±	1.30	1.90	±	0.70	32.6	±	6.2	1.80	±	0.30
12 - Della Calce	3.34	±	0.40	0.17	±	0.03	11.60	±	1.70	1.70	±	0.50	42.8	±	0.3	3.40	±	0.50
13 - Della Recca	2.56	±	0.09	0.08	±	0.01	8.20	±	0.90	2.50	±	0.00	25.5	±	5.8	2.30	±	0.10
14 - Don Vincenzo	2.94	±	0.17	0.21	±	0.02	8.70	±	0.20	2.10	±	0.50	33.4	±	1.4	2.80	±	0.20
15 - Imperatore	1.76	±	0.38	0.24	±	0.01	12.30	±	0.20	2.50	±	0.70	43.7	±	6.8	2.30	±	0.30
16 - Imperiale	3.77	±	0.73	0.18	±	0.01	2.90	±	0.60	13.0	±	0.70	56.2	±	12.7	1.10	±	0.00
17 - Lattacci	2.77	±	0.38	0.14	±	0.02	14.80	±	0.50	1.50	±	1.00	56.4	±	8.6	1.70	±	0.10
18 - Lauretana	2.86	±	0.09	0.31	±	0.06	13.60	±	2.80	2.00	±	0.00	35.7	±	6.2	0.40	±	0.00
19 - Limoncella	2.52	±	0.22	0.18	±	0.02	12.00	±	0.90	3.10	±	0.40	89.0	±	2.6	1.00	±	0.00
20 - Maggiaiolella	2.15	±	0.31	0.24	±	0.02	14.60	±	0.70	2.00	±	0.70	54.2	±	2.0	0.80	±	0.00
21 - Maiatica di Taurasi	2.24	±	0.10	0.22	±	0.01	6.90	±	0.50	2.80	±	0.70	39.2	±	9.0	1.40	±	0.10
22 - Marfatana	2.53	±	0.41	0.14	±	0.02	8.80	±	0.30	3.00	±	0.50	84.7	±	4.3	1.80	±	0.40
23 - Melella	2.13	±	0.21	0.12	±	0.02	1.40	±	0.20	6.60	±	0.30	21.9	±	5.5	1.70	±	0.20
24 - Montenero	3.54	±	0.47	0.31	±	0.05	1.20	±	0.00	1.70	±	0.10	34.8	±	2.8	1.80	±	0.20
25 - Mulegnana Nera	2.19	±	0.28	0.60	±	0.01	2.50	±	0.50	2.20	±	0.50	53.9	±	9.3	2.30	±	0.10
26 - Mulegnana Riccia	2.41	±	0.20	0.14	±	0.02	6.40	±	0.60	1.30	±	0.00	35.9	±	7.9	1.90	±	0.30
27 - Murana	7.62	±	1.06	0.18	±	0.03	14.60	±	2.00	3.60	±	0.30	32.5	±	0.9	1.90	±	0.20
28 - Nera dura di Mugnano	2.70	±	0.55	0.13	±	0.01	4.60	±	0.20	2.00	±	0.30	101.0	±	19.3	3.90	±	0.40
29 - Paesanella	2.56	±	0.23	0.14	±	0.02	6.20	±	0.60	2.40	±	0.00	87.2	±	17.5	1.60	±	0.00
30 - Pagliaccio	2.74	±	0.55	0.60	±	0.09	0.70	±	0.00	1.00	±	0.20	8.9	±	1.3	1.20	±	0.20
31 - Pagliarella	2.11	±	0.20	0.18	±	0.03	13.40	±	2.70	4.20	±	0.10	44.2	±	5.7	2.00	±	0.30
32 - Passaguai	3.10	±	0.27	0.25	±	0.03	5.90	±	0.90	0.40	±	0.00	54.5	±	1.8	2.90	±	0.50
33 - Patanara	2.09	±	0.30	0.21	±	0.00	13.50	±	0.40	3.70	±	0.50	27.9	±	4.3	2.30	±	0.40
34 - Pomella	3.86	±	0.37	0.22	±	0.01	6.00	±	0.10	5.30	±	0.60	22.4	±	1.4	1.60	±	0.30
35 - Regina	3.11	±	0.41	0.42	±	0.07	0.40	±	0.00	4.60	±	0.50	55.9	±	8.3	2.00	±	0.30
36 - San Michele	3.81	±	0.36	0.30	±	0.01	20.50	±	1.60	1.40	±	0.20	83.3	±	4.6	1.80	±	0.20
37 - Santa Teresa	2.65	±	0.45	0.22	±	0.02	4.50	±	0.50	3.90	±	1.00	57.7	±	4.1	1.30	±	0.20
38 - Sant'Anna	2.25	±	0.21	0.12	±	0.01	19.10	±	0.50	1.70	±	0.50	34.9	±	6.7	2.20	±	0.10
39 - Sbarbato	2.14	±	0.42	0.15	±	0.01	6.10	±	1.20	4.00	±	0.70	32.6	±	8.0	0.80	±	0.10
40 - Silvestre	2.66	±	0.45	0.31	±	0.01	11.6	±	0.90	2.30	±	0.50	26.6	±	2.7	1.40	±	0.20
41 - Spernocchia	3.26	±	0.33	0.23	±	0.02	5.10	±	0.00	2.60	±	0.00	29.3	±	2.0	1.70	±	0.20
42 - Tamburella	3.68	±	0.80	0.17	±	0.02	2.80	±	0.10	11.70	±	2.60	67.1	±	5.5	2.10	±	0.20
43 - Zuccarenella	7.72	±	0.15	0.13	±	0.01	10.10	±	0.40	2.20	±	0.00	44.8	±	8.7	2.00	±	0.50
44 - Bigarreaux	2.19	±	0.18	0.21	±	0.02	7.70	±	0.30	1.00	±	0.30	33.0	±	1.6	2.50	±	0.30
45 - Del Monte	2.40	±	0.26	0.18	±	0.02	7.70	±	0.60	2.70	±	0.50	57.1	±	9.5	1.60	±	0.30
46 - Ferrovia	2.04	±	0.33	0.28	±	0.02	7.50	±	0.60	1.20	±	0.00	37.5	±	7.4	1.50	±	0.30

**Table 2:** Tocopherols, total polyphenols and anthocyanins analysed in sweet cherry fruits. Tocopherols are expressed as mg 100 g^-1^ FW ± SD (n=3). Total polyphenols and anthocyanins are expressed as µg 100 g^-1^ FW ± SD (n=3).

**Accession**	**Total tocopherols**			**α-tocopherol**			**γ-tocopherol**			**Total polyphenols**			**Anthocianins**		
1 - Antuono	283	±	32	189	±	34	94	±	4	90	±	6	39	±	4
2 - Bertiello	223	±	42	106	±	21	117	±	27	124	±	1	6	±	1
3 - Bologna	209	±	33	137	±	20	72	±	13	68	±	5	6	±	0
4 - Camponica	319	±	3	199	±	10	121	±	7	105	±	7	6	±	1
5 - Cannamela	238	±	23	133	±	5	105	±	17	111	±	5	19	±	3
6 - Casanova	242	±	36	172	±	20	71	±	18	86	±	4	43	±	3
7 - Cervina	475	±	81	229	±	31	246	±	50	261	±	17	12	±	1
8 - Cervone	364	±	31	253	±	13	112	±	18	190	±	11	3	±	0
9 - Ciauzara	213	±	13	130	±	9	83	±	5	119	±	8	3	±	1
10 - Corniola	216	±	38	106	±	19	110	±	23	69	±	4	5	±	0
11 - Corvina	303	±	30	215	±	23	88	±	7	75	±	1	17	±	2
12 - Della Calce	276	±	30	204	±	22	72	±	22	63	±	4	2	±	0
13 - Della Recca	197	±	22	124	±	11	73	±	11	86	±	3	2	±	0
14 - Don Vincenzo	419	±	29	174	±	22	246	±	7	93	±	6	14	±	1
15 - Imperatore	179	±	1	119	±	2	60	±	3	71	±	3	4	±	0
16 - Imperiale	195	±	2	132	±	13	63	±	10	165	±	11	4	±	0
17 - Lattacci	232	±	22	157	±	21	75	±	3	94	±	6	4	±	0
18 - Lauretana	282	±	0	170	±	0	112	±	0	111	±	7	12	±	1
19 - Limoncella	288	±	27	203	±	31	85	±	18	176	±	8	3	±	0
20 - Maggiaiolella	354	±	10	158	±	13	196	±	15	213	±	13	13	±	2
21 - Maiatica di Taurasi	289	±	65	173	±	36	116	±	30	111	±	4	14	±	1
22 - Marfatana	149	±	30	115	±	14	34	±	17	46	±	1	3	±	0
23 - Melella	235	±	15	138	±	33	98	±	18	109	±	6	2	±	0
24 - Montenero	224	±	41	151	±	36	74	±	13	48	±	3	34	±	3
25 - Mulegnana Nera	290	±	39	186	±	30	104	±	12	88	±	5	4	±	0
26 - Mulegnana Riccia	283	±	26	171	±	26	112	±	0	116	±	6	64	±	2
27 - Murana	295	±	34	208	±	38	87	±	19	242	±	14	12	±	2
28 - Nera dura di Mugnano	191	±	45	109	±	21	82	±	25	102	±	7	23	±	2
29 - Paesanella	213	±	37	123	±	15	90	±	22	61	±	4	3	±	0
30 - Pagliaccio	396	±	13	285	±	13	112	±	0	52	±	4	27	±	1
31 - Pagliarella	393	±	36	247	±	20	146	±	16	88	±	5	3	±	0
32 - Passaguai	325	±	33	158	±	19	167	±	17	110	±	0	13	±	0
33 - Patanara	306	±	77	174	±	33	132	±	44	87	±	5	1	±	0
34 - Pomella	271	±	14	116	±	17	155	±	8	112	±	0	20	±	3
35 - Regina	342	±	41	223	±	13	119	±	32	110	±	0	13	±	0
36 - San Michele	726	±	145	310	±	37	416	±	115	164	±	11	1	±	0
37 - Santa Teresa	399	±	73	162	±	38	238	±	36	193	±	13	11	±	0
38 - Sant'Anna	269	±	13	186	±	10	83	±	20	75	±	3	14	±	1
39 - Sbarbato	309	±	50	182	±	30	127	±	25	110	±	0	13	±	0
40 - Silvestre	229	±	14	172	±	14	57	±	1	63	±	3	35	±	0
41 - Spernocchia	301	±	48	173	±	18	128	±	32	57	±	4	27	±	4
42 - Tamburella	226	±	18	136	±	5	90	±	14	164	±	2	6	±	1
43 - Zuccarenella	174	±	24	109	±	24	65	±	0	62	±	3	2	±	0
44 - Bigarreaux	398	±	61	229	±	57	169	±	4	110	±	0	13	±	0
45 - Del Monte	188	±	3	124	±	12	64	±	14	102	±	10	11	±	1
46 - Ferrovia	246	±	43	150	±	14	96	±	29	110	±	0	13	±	0

In Table 3 the activities of enzymes involved in antioxidant system were considered: ascorbate peroxidase (APX; EC 1.11.1.11), and the soluble and bound forms of polyphenol oxidase (PPO; EC 1.10.3.1) and guaiacol peroxidase (POD; EC 1.11.1.7). In particular, POD and PPO, in addition to acting as ROS scavengers, are also responsible for the oxidation of phenolic compounds to quinones, in presence of O_2_ (PPO) or H_2_O_2_ (POD), and their subsequent, polymerization to melanin. This phenomenon known as enzymatic browning, that occurs during fruit storage [6] negatively affects fruit color, taste, flavor, nutritional value and consistence and causes more than 50% of fruit losses [7].

**Table 3:** Enzymatic activities measured in analysed sweet cherry fruits. PODs and PPOs activities are expressed as µmol min^-1^ g^-1^FW; APX activity is expressed as nmol min^-1^ g^-1^FW.

**Accession**	**soluble POD**			**bound POD**			**soluble PPO**			**bound PPO**			**APX**		
1 - Antuono	2.08	±	0.28	1.86	±	0.33	0.35	±	0.05	0.08	±	0.01	1.84	±	0.33
2 - Bertiello	1.72	±	0.24	1.73	±	0.20	0.57	±	0.04	0.11	±	0.02	2.04	±	0.05
3 - Bologna	2.76	±	0.19	2.04	±	0.08	1.40	±	0.20	0.34	±	0.02	1.06	±	0.15
4 - Camponica	2.24	±	0.30	1.64	±	0.27	0.27	±	0.06	0.08	±	0.01	1.95	±	0.44
5 - Cannamela	3.21	±	0.31	1.70	±	0.25	0.54	±	0.01	0.10	±	0.01	1.88	±	0.14
6 - Casanova	2.06	±	0.14	1.22	±	0.06	0.27	±	0.02	0.04	±	0.00	2.88	±	0.36
7 - Cervina	2.01	±	0.30	1.00	±	0.12	0.26	±	0.01	0.04	±	0.00	1.77	±	0.20
8 - Cervone	2.58	±	0.22	2.02	±	0.06	0.22	±	0.00	0.02	±	0.00	1.90	±	0.07
9 - Ciauzara	2.91	±	0.35	0.73	±	0.04	0.17	±	0.01	0.12	±	0.02	1.33	±	0.28
10 - Corniola	3.24	±	0.28	0.86	±	0.10	0.23	±	0.01	0.15	±	0.00	0.68	±	0.14
11 - Corvina	2.31	±	0.13	2.28	±	0.27	0.39	±	0.00	0.07	±	0.02	1.65	±	0.35
12 - Della Calce	2.51	±	0.29	2.17	±	0.22	0.28	±	0.05	0.03	±	0.00	1.52	±	0.10
13 - Della Recca	2.10	±	0.22	0.79	±	0.08	0.21	±	0.01	0.15	±	0.00	1.62	±	0.10
14 - Don Vincenzo	1.72	±	0.20	0.76	±	0.05	0.12	±	0.00	0.13	±	0.02	1.97	±	0.07
15 - Imperatore	0.74	±	0.14	1.33	±	0.13	0.50	±	0.05	0.32	±	0.01	1.39	±	0.22
16 - Imperiale	1.65	±	0.13	1.17	±	0.01	0.34	±	0.07	0.03	±	0.00	2.70	±	0.29
17 - Lattacci	3.05	±	0.32	2.33	±	0.07	0.21	±	0.02	0.08	±	0.01	1.38	±	0.23
18 - Lauretana	3.79	±	0.32	2.63	±	0.26	0.31	±	0.07	0.05	±	0.00	2.65	±	0.18
19 - Limoncella	0.66	±	0.08	3.68	±	0.82	0.10	±	0.02	0.05	±	0.01	1.33	±	0.28
20 - Maggiaiolella	0.71	±	0.14	3.61	±	0.42	0.05	±	0.00	0.08	±	0.02	1.49	±	0.11
21 - Maiatica di Taurasi	3.03	±	0.19	2.22	±	0.46	0.46	±	0.08	0.05	±	0.01	1.51	±	0.06
22 - Marfatana	4.66	±	0.23	0.68	±	0.12	0.22	±	0.03	0.06	±	0.01	0.50	±	0.10
23 - Melella	1.79	±	0.04	3.45	±	0.26	0.75	±	0.18	0.26	±	0.03	2.85	±	0.29
24 - Montenero	1.74	±	0.11	0.78	±	0.07	0.20	±	0.02	0.06	±	0.01	1.08	±	0.05
25 - Mulegnana Nera	0.80	±	0.04	1.41	±	0.02	0.10	±	0.01	0.28	±	0.03	1.03	±	0.07
26 - Mulegnana Riccia	2.50	±	0.13	0.90	±	0.05	0.19	±	0.01	0.14	±	0.02	1.23	±	0.29
27 - Murana	0.37	±	0.02	3.96	±	0.65	0.04	±	0.01	0.09	±	0.00	1.07	±	0.13
28 - Nera dura di Mugnano	2.92	±	0.23	0.92	±	0.07	0.26	±	0.01	0.08	±	0.02	0.57	±	0.02
29 - Paesanella	3.14	±	0.37	0.99	±	0.18	0.22	±	0.01	0.08	±	0.00	0.61	±	0.11
30 - Pagliaccio	2.51	±	0.10	0.72	±	0.11	0.11	±	0.02	0.10	±	0.01	0.18	±	0.01
31 - Pagliarella	3.27	±	0.16	3.10	±	0.34	1.18	±	0.01	1.03	±	0.06	1.48	±	0.02
32 - Passaguai	1.40	±	0.07	2.52	±	0.14	0.26	±	0.03	0.05	±	0.01	1.98	±	0.20
33 - Patanara	1.80	±	0.28	2.11	±	0.09	0.91	±	0.17	0.78	±	0.01	1.48	±	0.32
34 - Pomella	1.38	±	0.28	0.81	±	0.13	0.21	±	0.02	0.10	±	0.01	0.85	±	0.16
35 - Regina	0.52	±	0.02	0.80	±	0.05	0.16	±	0.03	0.14	±	0.01	1.69	±	0.20
36 - San Michele	1.37	±	0.34	3.10	±	0.05	0.35	±	0.05	0.07	±	0.00	1.89	±	0.22
37 - Santa Teresa	0.92	±	0.07	2.67	±	0.42	0.82	±	0.16	0.04	±	0.01	1.47	±	0.08
38 - Sant'Anna	3.02	±	0.36	1.86	±	0.02	0.51	±	0.07	0.04	±	0.01	1.48	±	0.31
39 - Sbarbato	1.34	±	0.16	1.27	±	0.05	0.38	±	0.11	0.04	±	0.00	2.73	±	0.35
40 - Silvestre	1.88	±	0.08	0.62	±	0.09	0.26	±	0.05	0.11	±	0.02	1.60	±	0.35
41 - Spernocchia	2.08	±	0.14	1.43	±	0.12	0.34	±	0.03	0.04	±	0.01	1.39	±	0.19
42 - Tamburella	1.36	±	0.03	3.68	±	0.40	0.33	±	0.00	0.19	±	0.01	3.10	±	0.20
43 - Zuccarenella	3.37	±	0.02	1.31	±	0.13	0.28	±	0.01	0.11	±	0.01	1.49	±	0.01
44 - Bigarreaux	2.38	±	0.08	1.37	±	0.06	0.39	±	0.08	0.04	±	0.01	2.32	±	0.34
45 - Del Monte	1.04	±	0.23	1.94	±	0.15	0.92	±	0.05	0.41	±	0.06	0.64	±	0.13
46 - Ferrovia	1.76	±	0.01	1.38	±	0.02	0.31	±	0.08	0.04	±	0.01	2.14	±	0.32

## 2 Experimental design, materials and methods

To identify the accessions which better resist to oxidative stress and, therefore, are more suitable for the long-term storage, sweet cherry fruits of forty-three accessions from Campania region were harvested and collected at commercial maturity, in the regional experimental farm “Improsta”, Eboli Campania, Italy (approximately at 14°58′E, 40°33′N). One additional commercial accession (Bigarreaux) and two commercial cultivars (Del Monte and Ferrovia) were used as reference. The data were obtained by biological triplicates, each one constituted by twenty fruits from five plants, analyzed separately. The fleshy part of fruits was cut, frozen in liquid nitrogen, and powdered [8]as soon as the samples arrived in the laboratory and then stored at -80°C for preserving the antioxidant enzymatic activities and antioxidant metabolite levels.

Reduced and oxidized ascorbate (AsA+DHA) and reduced and total (reduced plus oxidized) glutathione (GSH) were extracted and evaluated as described in Annunziata et al. [9]. Tocopherols determination was performed by HPLC, modifying the method described by Annunziata et al. [9]. Tocopherols were determined measuring their fluorescence with excitation at 295 nm and emission at 340 nm, and the concentration was determined for comparison with standard curves of pure α-, or γ-tocopherol in the range 0.06–2 mg L^-1^. Hydrogen peroxide (H_2_O_2_) malondialdehyde (MDA) were assayed according to Woodrow et al. [4]. Total phenolics were measured by the Folin–Ciocalteu method of Ainsworth and Gillespie [10]. Anthocyanins were assayed by UV-Visible spectroscopy method of Giusti and Wrolstad [11].

Enzyme activity analyses were performed by spectrophotometer assays. Polyphenol oxidase (PPO; EC 1.10.3.1) and guaiacol peroxidase (POD; EC 1.11.1.7) were simultaneously extracted by a two-step extraction protocol, that allowed the separation of both soluble and membrane bound isoforms [1]. POD activities were measured in excess of *o*-tolidine, used as substrate. PPO analysis was performed using catechol as substrate. Ascorbate peroxidase (APX; EC 1.11.1.11) was extracted and assayed according to Hediye Sekmen et al. [12].
